# Obesity and diabetic retinopathy: What is the association?

**Published:** 2015

**Authors:** Behzad Heidari

**Affiliations:** 1 Mobility Impairment Research Center, Babol University of Medical Sciences, Babol, Iran; 2 Department of Internal Medicine, Ayatollah Rouhani Hospital, Babol University of Medical Sciences, Babol, Iran.

Diabetes type 2 is one of the most prevalent causes of morbidity and mortality in developed countries (1). Current epidemic of diabetes across various geographic regions of the world including Iran is linked to high prevalence rates of obesity, metabolic syndrome involving all age groups even young and children due to changes in eating habits, low physical activities and lifestyles (2-6). Diabetes in itself results in several complications and diabetic retinopathy (DR) is one of the most common complications and an important cause of visual loss and disability. 

The prevalence rate of DR varies across diverse populations in hospital-based diabetic patients is expected to be higher than patients recruited in population based-studies (7-11). In a prospective study of general population in China, over a 4-year follow-up period, the incidence of DR was 1.81% person-year (7). In another population-based study, aged 35-74 years, the prevalence of DR was 21.7% (9). In a cross-sectional study of Saudi patients with type 2 diabetes aged ≥ 25 years, the overall prevalence of DR was 19.7% (8). In a large cohort of diabetic patients in Germany, over 13-years, DR was present in 20.12% (10). In another study of diabetic patients (types 1 and 2), aged 18-70 years, the prevalence of DR was 64% (11). 

Similarly, the associated factors of DR also is anticipated to be different across various studies with regard to age of patients, duration of diabetes, the status of diabetes control and the prevalence of coexistent conditions like hypertension, hyperlipidemia, obesity in the general population (7-10). In this issue of the Caspian Journal of Internal Medicine, Rasoulinejad et al. (12) in a case -control study of 1562 patients with type 2 diabetes, with mean age of 54±10.6 years and mean diabetes duration of 9.6±7 years have found DR in 64.1%. The study population was referred to a teaching hospital for evaluation of DR. The results of the study demonstrated a positive association of age, duration of diabetes, family history of diabetes, poor diabetes control, nephropathy with DR. 

 Whereas, dyslipidemia, smoking and hypertension were not associated with DR and unexpectedly, BMI was negatively associated with DR (12). In earlier studies, several factors like age, duration of diabetes, hypertension (HTN), smoking, gender, obesity, family history of diabetes, dyslipidemia, poor control of diabetes, insulin use, neuropathy and nephropathy were shown to be associated with DR. A number of these factors were also associated with the development of DR in diabetic patients without diabetes (1, 7-10, 13). The results of the aformentioned study agree with the reports of many previously published studies (7-10), but differ with a number of studies regarding lack of association between HTN, dyslipidemia and particularly obesity with DR (9, 10, 13). Most related studies have found an association between obesity and DR (7-10) and thus, the results of this study in relation to BMI and DR require further consideration. Due to the geographic region of this study, obesity and associated factors of obesity are highly prevalent (2-6). The negative association may be attributed to patient selection. However, as the authors studied only the patients who presented with ocular problem, so many diabetic patients without DR as well as patients with undiagnosed DR were not included. Therefore, the study patients are not representative of diabetic patients in the general population and the real number of patients with DR is expected to be overestimated. In addition, the diabetic patients with and without DR were compared based on BMI value and were not classified according to the presence or absence of obesity. Lower BMI in patients with DR can be attributed to more severe disease, poor diabetes control and possibly greater weight loss as compared with patients without DR. Thus, lower BMI in DR group may be attributed to more severe disease rather than DR. 

Obesity dose not only play a pivotal role in the development of diabetes and DR, but also is an important factor for the development of many prevalent conditions like HTN and its ensuing cardiovascular and cerebrovascular complications, osteoarthritis, dyslipidemia and metabolic syndrome (13, 14). Available data indicate a relationship between obesity, type 2 diabetes, and cardiovascular disease with inflammation (15, 16). In a study of diabetic patients, increasing BMI was associated with increased risk of complications (13). The results of a systematic review and meta-analysis of 51 cross-sectional studies showed a positive correlation between C-reactive protein (CRP) and obesity as assessed by waist circumference, BMI or waist to hip ratio (17). Serum CRP is a known marker of inflammation and a predictor of future development cardiovascular diseases (18, 19). In a study of obese diabetic patients there was a relationship between waist circumference and serum CRP levels suggesting an association between obesity and inflammation (20). In diabetic patients, increasing high sensitive CRP levels correlated with severity of DR and the association was more pronounced in patients with BMI ≥ 30 kg/m 2 indicating that obesity imposes diabetic patients to more severe DR due to inflammatory process (11). Both obesity and inflammation have a role in the development of endothelial impairment involved in DR (21). 

The pathogenesis of DR is complex and several mechanisms including vascular, inflammatory and neuronal mechanisms are responsible in mediating structural and molecular alterations in DR (22). Contribution of any associated factors in the development of DR in addition to severity of diabetes, and the status of diabetes control is partly dependent to the prevalence of these factors in the general population. In diabetic patients recruited from general population living in the North of Iran, prevalence of obesity, diabetes, HTN, hyperlipidemia, and metabolic syndrome, is high and thus identifying the contribution of any responsible factors requires a long-term prospective longitudinal study.
